# Bax- Bcl-xL interaction dynamics during the progression of cell cycle and cell death using FLIM-FRET

**DOI:** 10.15698/cst2025.07.307

**Published:** 2025-07-08

**Authors:** Aman M. Halikar, Aneesh Chandrasekharan, Asha Lekshmi, Aswathy Sivasailam, Jain Tiffee P J, Shivanshu K. Tiwari, Aijaz Ahmad Rather, TR Santhoshkumar

**Affiliations:** 1 Cancer Research Program, Rajiv Gandhi Centre for Biotechnology, Poojappura, Thycaud P.O., Thiruvananthapuram, Kerala 695014, India.; 2 Manipal Academy of Higher Education (MAHE), Manipal, Karnataka-576104, India.

**Keywords:** FLIM-FRET, protein-protein interactions, apoptosis, Bax, Bcl-xL

## Abstract

Genetically identical cells in a population show cell-to-cell variability because of fluctuation in transcription, epigenetics, post-translational modifications, and stochastic or extrinsically triggered non-genetic alterations. The change in the interaction state of proteins also emerges as an additional layer of cell signaling that influences cell cycle and cell death. However, the interrelation between cell cycle progression and cell death under the influence of spatio-temporal changes in protein-protein interaction is difficult to demonstrate in growing cells. This requires tools for cell cycle phase-resolved visualization of macromolecular interactions in live cells. We describe an approach to visualize the interaction of pro- and anti-death signaling partners, Bax and Bcl-xL, during cell cycle progression and cell death in live cells. Cells were stably expressed with Bax and Bcl-xL with FRET pairs and real-time cell cycle indicator probes. Acceptor photobleaching and Fluorescence lifetime imaging revealed interaction dynamics between Bax and Bcl-xL in isogenic stable cells. While Bcl-xL inhibited cell cycle progression, Bax promoted the cell cycle. The study highlighted an increased Bax-Bcl-xL interaction in the G1 phase compared to the non-G1 phase. Increased interaction is seen under stressed conditions and Bax-activated cells with FLIM-FRET, highlighting the nature of Bax-Bcl-xL interaction during cellular stress. In conclusion, our study explains Bax-Bcl-xL interaction dynamics in real-time and the potential utility of the approach to study macromolecular interactions along with cell cycle and cell death.

## Abbreviations

FLIM-FRET – Fluorescence lifetime imaging,

FRET – Forster resonance energy transfer,

KO – Kusabira orange.

## INTRODUCTION

Apoptosis is a tightly regulated process that plays an essential role in development, tissue homeostasis, and disease. Various stimuli initiate apoptosis, including DNA damage, cellular stress, and death receptor signaling 1,2,3. Once triggered, apoptosis is executed by a cascade of proteases that cleave cellular proteins and organelles, leading to cell death. Cell progression toward cell death and its interrelation with the cell cycle have been studied widely 4,5,6. Studies have shown the importance of crucial checkpoints (G1/S, G2/M, SAC) and the regulatory proteins critical in the inevitable shift of cells towards arrest and subsequent apoptosis. B-cell lymphoma-extra-large (Bcl-xL) is known to slow down the G1/S transition in cells, thereby controlling both proliferation and apoptosis 7,8. On the other hand, pro-apoptotic protein Bcl2-associated agonist of cell death (Bad) is shown to accelerate cells through the cell cycle, underscoring their compensation for each other in the cell cycle 8.

Among the pro-apoptotic members, Bax is a pore former that plays a pivotal role in triggering apoptosis, while Bcl-xL is an anti-apoptotic member known for its ability to inhibit apoptosis 9,10,11. Both possess all four BH domains, including a BH3 domain essential for apoptotic function. Activator BH3-only proteins (BID/BIM) bind Bax and trigger its accumulation on the mitochondrial membrane 12. This leads to the homo-oligomerization of Bax and the pore formation on the outer mitochondrial membrane (MOMP), triggering the release of Cytochrome C 9,13. According to the prevalent model, preferential binding of Bcl-xL with Bax on mitochondria and its subsequent retrotranslocation into the cytosol prevents apoptotic cell death in cancer cells 10.

Previous studies using single-cell imaging and computational methods, single-cell multi-omics, etc., have revealed complex cell-to-cell variabilities as a source of the differential response of cancer cells to anticancer drugs 14. However, a confirmatory role for the cell cycle in regulating apoptotic stress response is yet to be generated, even though alterations of many transcripts are implicated in both the cell cycle and cell death decisions, such as Bax and Bcl-xL 15,16. It is a fact that many proteins' functions within the cells are governed not only by transcript density but also by their localization and interaction partners. Supporting this, a recent study using proteome-wide cellular thermal shift assay revealed changes in interaction states for more than 750 proteins during the cell cycle 17. This indicates that an additional layer of regulation is possible at the macromolecular interaction level that could act as a source of variability that needs to be identified at the single-cell level. It requires rigorous methodological approaches from real-time and large-scale drug-cell studies to confirm the generality. Traditional methods such as co-immunoprecipitation and immunofluorescence have provided valuable insights into the static nature of protein-protein interactions at the population level. However, they are limited in their ability to capture the dynamic nature of these interactions in real-time at a single-cell level. Real-time visualization techniques, such as Forster resonance energy transfer (FRET) and FLIM-FRET (Fluorescence lifetime imaging), have emerged as powerful tools for studying protein-protein interactions in living cells 18,19. FLIM-FRET employs the principle of FRET, where donor excitation results in the non-radiative transfer of energy from donor to acceptor through a dipole-dipole interaction, thus resulting in the reduction of the donor's fluorescence lifetime 20. FLIM-FRET is particularly valuable as it can provide the quantitative information that is often difficult to obtain from intensity-based FRET imaging alone, as the former can yield information on the interaction strength between proteins and the relative distance in different cellular compartments. FRET-based PPI study and visualization of the cell cycle using real-time probes such as FUCCI (Fluorescent ubiquitination-based cell cycle indicator) is yet to be demonstrated for understanding such complex signaling. Appropriate fluorescent combinations of FUCCI partners are required to couple with fluorescent FRET pairs. The first generation FUCCI employed Geminin ( Azami green ) - an inhibitor of DNA replication licensing activity present in S, and CDT1, a licensing factor for DNA replication in G1, tagged with Kusabira orange (KO) 21. Recently, miRFP 670 geminin with 640Ex/670Em was described 22. By employing FRET-based probes of ECFP-YFP that specifically detect the interaction between Bax and Bcl-xL, we have developed a FUCCI probe with red fluorescent mKO2-hCdt1(30/120) and miRFP geminin to analyze the dynamic changes in their interaction during various phases of the cell cycle and under conditions of cell death.

The study confirmed that Bax-Bcl-xL interaction is spatiotemporally regulated in cycling cells and shows a stronger skew towards the G1 phase of the cell cycle, and the heterogeneity of interaction was visible between cells. YFP Bcl-xL overexpressing cells showed slower cell cycle progression with longer G1/S phase, but ECFP Bax co-expression accelerated the progression. Notably, strong Bax-Bcl-xL interaction was observed even in Bax-activated cells where Bax accumulated on mitochondria, presumably for pore formation during cell stress. Our findings provide new insights into the regulation of Bax-Bcl-xL interaction heterogeneity during cell cycle progression and cell death. These findings and approaches described here could be used to develop new strategies for studying protein-protein interactions across a wide range of proteins and to address potential roles in cell-to-cell response heterogeneity.

## RESULTS

### Stable expression of ECFP Bax, YFP Bcl-xL, and ECFP Bax-YFP Bcl-xL and its functional validation

**Figure 1:**
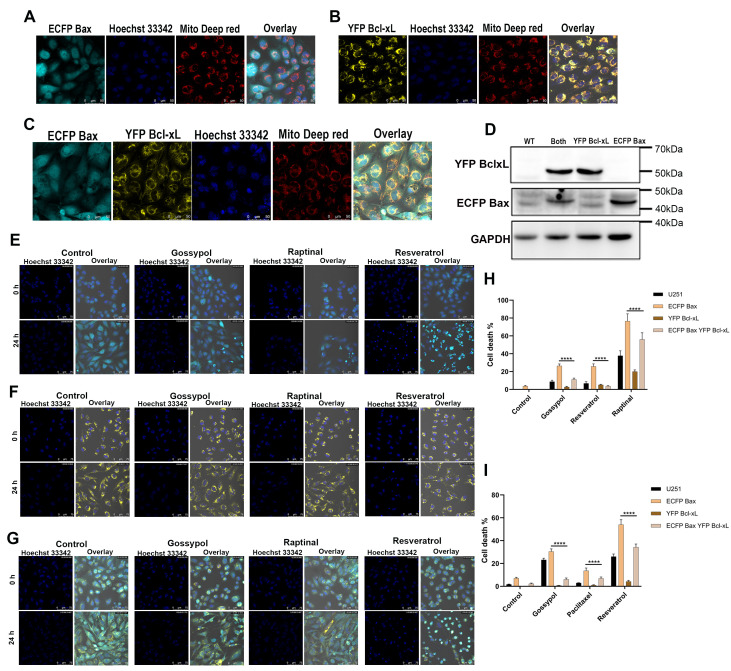
FIGURE 1: Cell line development and functional validation of ECFP Bax, YFP Bcl-xL, and ECFP Bax-YFP Bcl-xL cells. U251-MG Glioblastoma cells co-transfected with ECFP Bax, YFP Bcl-xL FRET probes, stained with Mito-Tracker Deep Red and Hoechst 33342. **(A)** Shows homogenous expression of ECFP Bax stable cells, **(B)** represents YFP Bcl-xL only stable cells. **(C) **shows stable cells expressing both ECFP Bax and YFP Bcl-xL; further validation was performed with immunoblots shown in **(D) **probing for ECFP Bax and YFP Bcl-xL at higher molecular weights 47 kDa and 57 kDa, respectively, (probed with Bax, Bcl-xL, and GAPDH). Functional validation was done with cell death imaging in Real-time with Hoechst 33342 used for cell death readout, and Annexin V FACS; **(E)** represents functional validation of ECFP Bax-only cells with different chemotherapeutic drugs against parental cells. Gossypol, Raptinal, and Resveratrol drugs were used at various concentrations; imaging showed higher Bax accumulation and cell death. YFP Bcl-xL only cells were similarly validated with the same compounds via imaging **(F)**, showing reduced cell death with stable Bcl-xL expression. Functional imaging validation of ECFP Bax-YFP Bcl-xL overexpressing cells is represented in **(G)**; statistical analysis of cell death imaging **(H)** was performed by counting dying cells in 24 h after treatment in time-lapse, experiments were repeated 3 times in multiple wells (n >100) **** p<0.0001 (one-way/two-way ANOVA). **(I)** shows Annexin cell death statistical analysis of all developed cells treated with chemotherapeutic drugs ****p<0.0001 (one-way ANOVA).

Since we were interested in understanding cell-to-cell interaction changes with cell cycle progression, we decided to have a monoclonal stable cell line model expressing both interacting partners using the best FRET donor and acceptor fluorescent protein pairs. To achieve this, we used the U251-MG glioblastoma cell line, known for its invasiveness and rapid clonal growth under *in-vitro* conditions. The cells were transfected with ECFP Bax as FRET donor and YFP Bcl-xL as FRET acceptor to get a stable single-cell colony expressing both the proteins, employing flow sorting and colony isolation. For flow sorting, 440/488 nm laser combinations were used, ECFP (Ex-440 nm) and YFP (Ex-488 nm), and the resulting stable cell overexpressing only ECFP Bax is represented in **Figure 1A**. The gating used for sorting and collecting stable cells is shown in Figure S1A. The YFP Bcl-xL-only expressing cells are represented in **Figure 1B**; cells overexpressing both proteins are shown in **Figure 1C. **The schematic illustration of methodologies employed to generate stable cells are represented in Figure S1C. Validation experiments for the protein overexpression were performed using western blotting (**Figure 1D**); the overexpressed proteins can be seen as higher molecular weight bands on the immunoblot of approximately 47 kDa and 57 kDa for ECFP Bax and YFP Bcl-xL, respectively. The overexpressed bands were probed with anti-Bax and Bcl-xL antibodies, respectively. Uncropped immunoblots are shown in Figure S2A. Lastly, the functional performance of stably expressing cells was validated via imaging after exposing the cells to diverse stresses such as Gossypol, Raptinal, Resveratrol, and Paclitaxel, where Hoechst 33342 was used to score chromatin

condensation as a readout of cell death. ECFP Bax cells showed rapid and higher cell death (**Figure 1E**), while the opposite was seen in YFP Bcl-xL overexpressing cells (**Figure 1F**). The results are consistent with the pro-apoptotic and anti-apoptotic function of Bax and Bcl-xL, respectively, highlighting stable expression of functional proteins. The pro-apoptotic response was balanced in ECFP Bax-YFP Bcl-xL cells, as both the proteins counteract each other, thus generating a balanced response (**Figure 1G**). The quantitative analysis of cell death was performed based on counting cells undergoing death at the end of 24 hours and converting them to cell percentages to normalize varying cell numbers, as shown in **Figure 1H**. We also confirmed the results using the Annexin V BFP binding assay by FACS. We have used Annexin V BFP to enable cell death analysis even in ECFP expressing cells with 355 nm laser excitation for BFP. As shown in **Figure 1I,** increased cell death was observed in ECFP Bax overexpressing cells. The YFP Bcl-xL-expressing cells demonstrated a reduction in annexin V positivity, confirming survival function (Figure S1B).

### Analysis of the interaction between ECFP Bax-YFP Bcl-xL(WT) and its mutants confirmed the interaction specificity between the proteins

**Figure 2:**
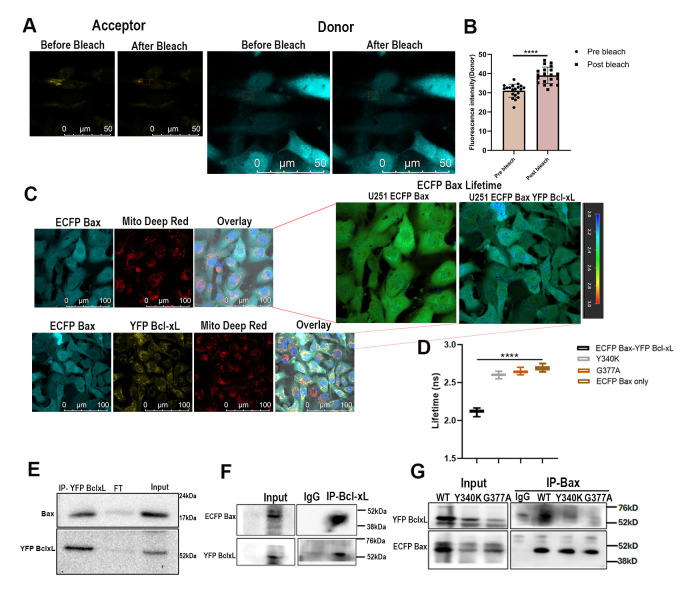
FIGURE 2: Analysis of ECFP Bax-YFP Bcl-xL interaction proves the functional utility of FRET probes in cells. ECFP Bax-YFP Bcl-xL cells were analyzed for FRET between ECFP Bax and YFP Bcl-xL using FRET AB **(A)**; donor and acceptor are represented as before and after bleach. The acceptor was bleached, and increased donor intensity was recorded in ROIs. A statistical representation of donor intensity increase **(B) **after FRET AB imaging**. **FLIM-FRET imaging was done with the donor only as a control, and both ECFP Bax-YFP Bcl-xL cells show ECFP lifetime reduction, indicating a strong interaction between proteins **(C) **the reduction in donor lifetime is indicative of FRET. **(D)** represents ECFP Bax lifetime analysis in co-expressing wildtype, Y340K, G377A mutants, and donor alone cells. Lifetime values showed a significant decrease, pointing towards a strong interaction in the FRET pair (n=32) ****p<0.0001 (unpaired two-tailed t-test). **(E)** GFP trap CO-IP was performed on YFP Bcl-xL only cells to pull the interaction complex of YFP Bcl-xL and endogenous Bax (probed with Bax and Bcl-xL)**. **Similarly, another Co-IP with protein G beads **(F)** was done to analyze the interaction between ECFP Bax and YFP Bcl-xL by pulling YFP Bcl-xL (probed with Bcl-xL). **(G) **ECFP Bax pulldown was attempted in co-expressing and Bcl-xL mutant FRET pairs, showing a robust interaction in wild-type cells and a negligible association in mutant cells. cells were probed with anti-Bax and anti-Bcl-xL antibodies.

The prevalent notion is that Bax and Bcl-xL interact primarily on the mitochondrial membrane; weaker interactions are seen in the cytosol. We tested this by performing acceptor bleaching FRET imaging, FLIM-FRET, and co-immunoprecipitation on the cells. Acceptor bleaching FRET was used to test the strength of the traditional FRET detection method for assessing the interaction between Bax and Bcl-xL. The bleach areas were decided using ROIs, and the acceptor was bleached to 10-20% of the original intensity. The bleaching showed a significant increase in the donor intensity post-bleach; this was performed in 4% paraformaldehyde fixed cells, since factors like protein diffusion to the bleached part affected the resulting donor intensity in live samples (**Figure 2A**). Overall, the results showed a significant increase in donor intensity after bleach, and a mean FRET efficiency value (E=10±3%) was noted from multiple experiments (**Figure 2B**). FLIM-FRET imaging is an integral analysis to measure the interactions between proteins and compare them with the AB FRET results. Since the interaction studies involving proteins require collision controls for confirming specificity, we generated two mutants (Y101K and G138A) in Bcl-xL, modified with an enhanced Venus at the N terminus, which resulted in mutations at Y340K and G377A. Both mutants and a Venus-Bcl-xL wildtype were independently transfected transiently in U251 ECFP Bax stable cells for interaction studies. The schematic explaining the transfection approach for mutants is shown in Figure S4A. The donor-only sample was used to calculate the lifetime of ECFP Bax, which was around 2.68±0.2 ns across the cells. On the other hand, both ECFP Bax-YFP Bcl-xL cells showed an ECFP Bax lifetime of around 2.2±0.2 ns in the cells measured in **Figure 2C, D**. On the other hand, mutants showed consistently higher lifetimes in all areas, despite high expression of Bax and Bcl-xL, with Y340K showing 2.60±0.2 ns and G377A showing 2.64±0.2 ns (**Figure 2D**)**. **The imaging data comparing lifetimes between wildtype and mutant FRET pairs is shown in **Figure S4B, C**. The lifetime values of the donors were used to calculate FRET Efficiency (E) in wildtype and mutant Bcl-xL, which further confirmed that mutants failed to associate with Bax. The interaction was confirmed by the GFP-Trap co-immunoprecipitation using magnetic beads. U251 YFP Bcl-xL cell lysate was used in pull-down where YFP Bcl-xL was trapped by beads, and both Bax and Bcl-xL were probed using anti-Bax and anti-Bcl-xL antibodies, respectively (**Figure 2E**); note that the Bax that was pulled was endogenous, emphasizing the binding of YFP Bcl-xL to Bax in the cells. Figure S2B shows raw immunoblots from the GFP trap experiment. To validate interaction between both proteins, we performed IP for YFP-Bcl-xL to co-immunoprecipitate ECFP Bax. An anti-Bcl-xL antibody was used for immunoprecipitation, and the resulting co-immunoprecipitated ECFP-Bax was seen, validating their interaction (**Figures 2F**, S2C). Lastly, we confirmed the interaction between the FRET pair and compared it with the mutant Bcl-xL expressing FRET pairs to invalidate the possibility of nonspecific binding. Protein G beads were used to pull down Bax in U251 ECFP Bax-YFP Bcl-xL and both mutant cells containing ECFP Bax-*mut*Venus-Bcl-xLY340K and ECFP Bax-*mut*Venus-Bcl-xLG377A using an anti-Bax antibody, followed by probing using an anti-Bcl-xL antibody; **Figure 2G **shows co-immunoprecipitated YFP Bcl-xL in wildtype against mutants showing negligible association, clearly indicating specificity of interaction. Figure S2D represents uncropped immunoblots of Co-immunoprecipitation.

### Bax and Bcl-xL demonstrate opposing Cell cycle regulatory functions upon release from cell synchronization in ECFP Bax-YFP Bcl-xL co-expressing cells

**Figure 3:**
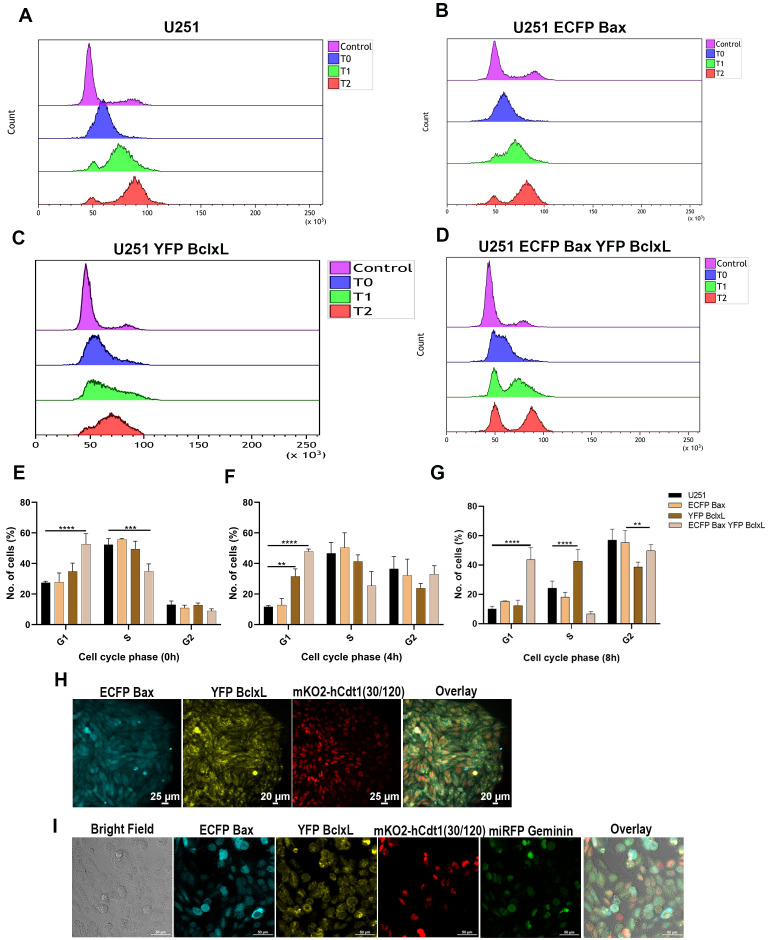
FIGURE 3: Cell cycle analysis of ECFP Bax, YFP Bcl-xL, and ECFP Bax-YFP Bcl-xL cells shows regulatory control by both proteins; Cell cycle FUCCI indicates accurate progression with oscillatory behavior in different cell cycle phases. Cell cycle analysis was carried out to evaluate cell cycle progression in cells; cells were synchronized and released; FACS analysis with Hoechst 33342 staining was done with a DAPI filter set at T0-0h, T1-4h, and T3-8h after release, respectively. The cells were analyzed using their respective and parental controls. **(A)** represents parental U251 cells at various time points, **(B)** represents ECFP Bax cells, **(C)** shows YFP Bcl-xL cells, and **(D)** shows ECFP Bax-YFP Bcl-xL cells analyzed at different time points after release from synchronization. Statistical analysis of cell cycle progression at 0hr **(E)**, Statistical analysis at 4hr is shown in **(F)**, and analysis of cell cycle progression at 8hr is represented in **(G)**. All statistical analyses were performed with (one-way/two-way ANOVA) *p<0.05 **** p<0.0001 (n > 10000). ECFP Bax-YFP Bcl-xL cells were transfected with mKO2-hCdt1(30/120), which was further transfected with miRFP Geminin. The cell colony showing the homogenous expression of mKO2-hCdt1(30/120) is represented in **(H)**; these stable mKO2-hCdt1(30/120) expressing cells were further transfected with miRFP Geminin **(I) **to generate stable cells showing both proteins' overexpression.

Even though Bax and Bcl-xL are well-known pro- and anti-apoptotic proteins, studies have emphasized their regulatory roles in the cell cycle 23. To understand the impact of the cell cycle regulatory role of ECFP Bax, YFP Bcl-xL, and both, cells were analyzed for cell cycle progression using the thymidine double block method. After the thymidine double block, cells were released from synchronization and analyzed at 4 h intervals. The results represented in **Figure 3E** show the cell cycle status immediately after block release as T0, **Figure 3F** and **Figure 3G** subsequently show T1(4 h) and T2(8 h), respectively. The release experiments confirmed the cell cycle regulatory functions of these proteins; ECFP Bax overexpressing cells showed rapid progression through the cell cycle (**Figure 3B**)**,** which was similar to the parental cells (**Figure 3A**), which can be clearly seen from analyses in **Figures 3E-G**. By the end of 8 h from release, U251 ECFP Bax cells show a similar number of cells in G2 as in control and significantly higher cells in G2 than in other cells (**Figure 3G**). The YFP Bcl-xL overexpressing cells showed prolonged G1S phase with the highest number of cells in the S phase by the end of 8 h, as shown in **Figure 3C**. The cell cycle of both ECFP Bax-YFP Bcl-xL overexpressing cells showed mostly equal distribution of both G1S and G2M cells by the end of the analysis, a characteristic that may have resulted from the overexpression of proteins with opposing activity, underscoring the role for Bax in the cell cycle in co-expressing conditions (**Figure 3D**). A representative flow cytometry histogram used for calculating cell phase is provided in **Figures S3B-E**. The *p*-values were calculated with one-way/two-way ANOVA and multiple comparisons t-tests to minimize error, and only significant dataset *p*-values were represented in the analyses to avoid confusion from data crowding.

Further, we developed stable cells with cell cycle indicator FUCCI probes; U251 ECFP Bax-YFP Bcl-xL cells were stably expressed with the G1-S cell cycle progression marker mKO2-hCdt1(30/120) and miRFP Geminin for S-G2-M, with the idea to image cell cycle progression and interaction changes in real-time. Additionally, ECFP Bax cells and YFP Bcl-xL cells were stably expressed with mKO2-hCdt1(30/120) to analyze cell cycle progression in these cells. Colony selection was used to get cells with stable expression of the mKO2-hCdt1(30/120). **Figure 3H** shows a stable mKO2-hCdt1(30/120) expressing colony with ECFP Bax-YFP Bcl-xL cells, which was used further to express the miRFP Geminin. The detailed FACS sorting ap-proach utilized to get the stable cells is represented in Figure S3A, showing the gating parameters. A schematic illustration of the strategy employed for developing FUCCI probes is shown in Figure S3F. The expression of fluorescent probes mKO2-hCdt1(30/120) and miRFP Geminin in ECFP Bax-YFP Bcl-xL FUCCI - FRET partner is shown in **Figure 3I**. Video S1 and video S2 show ECFP Bax and YFP Bcl-xL cells stably expressing mKO2-hCdt1(30/120). ECFP Bax-YFP Bcl-xL cells demonstrating the oscillation between mKO2-hCdt1(30/120) and miRFP Geminin with the progression of the cell cycle for 48 h are shown in video S3. The video further confirms that the cells stably expressed both FRET partners at a homogenous level with the functional real-time reporting of cell cycle progression probes.

### ECFP Bax-YFP Bcl-xL showed stronger interaction in the G1 phase of the cell cycle 

**Figure 4:**
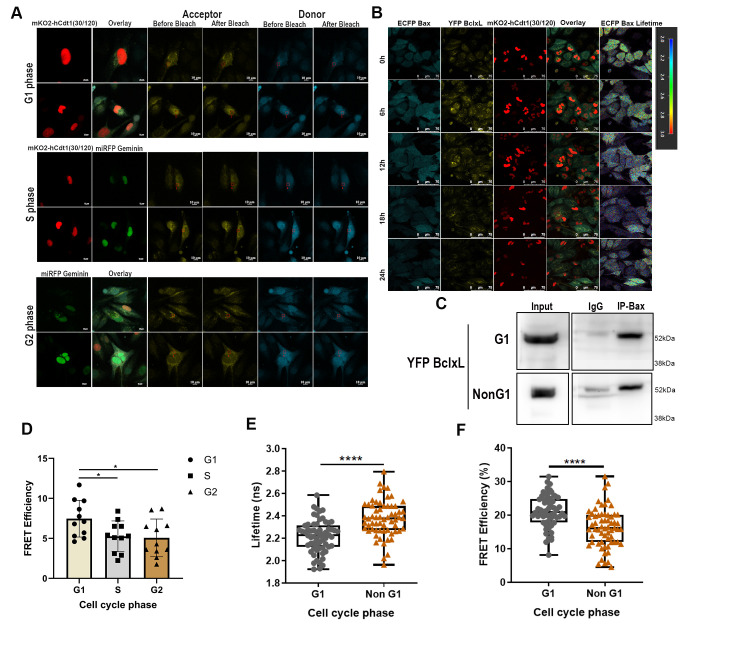
FIGURE 4: ECFP Bax-YFP Bcl-xL shows cell cycle-dependent interaction changes with stronger interaction in the G1 phase. Analysis of protein-protein interactions was done using FRET AB in the G1, S, and G2M phases of the cell cycle; cells in the S phase were marked by expression of both mKO2-hCdt1(30/120) and miRFP Geminin, **(A) **shows panels of G1, S, and G2 cells with acceptor and donor probes. A decrease in the acceptor after bleaching is shown as before bleach and after bleach, whereas an increase in the donor after bleach is shown in adjacent images; all ROIs are represented by red squares in the images. FLIM-FRET imaging of U251 ECFP Bax YFP Bcl-xL cells in real-time for 24 h showed variations in ECFP Bax lifetimes as seen in **(B)**; the cell cycle-dependent interaction changes were analyzed by different ECFP Bax cellular lifetimes recorded and analyzed separately for each cell; S-phase cells were not included to maintain sufficient sample number. Coimmunoprecipitation was done by harvesting cells in G1 and non-G1 phases after cell synchronization for ECFP Bax and YFP Bcl-xL interaction study; ECFP Bax was pulled using Bax antibody, and YFP Bcl-xL was probed with a Bcl-xL antibody **(C)**. Statistical analysis of FRET AB imaging done in different cell cycle phases showing strong interaction in G1 phase (n= 11) *p<0.05 (one-way ANOVA) **(D)**, analysis of cell cycle-dependent ECFP Bax lifetime imaging showing strong interaction in G1 phase **(E). **FRET efficiency analysis from FLIM-FRET imaging showing ECFP Bax-YFP Bcl-xL interaction variation across G1 and NonG1 cell cycle phases **(F)**, (n= 61) ****p<0.0001 (unpaired two-tailed t-test).

To test the interaction alterations between cell cycle phases, we performed acceptor bleaching FRET and FLIM-FRET experiments at a single-cell level based on cell cycle stage using the above validated cells. **Figure 4A** represents the acceptor bleaching experiment performed in cells of different cell cycle stages; the control was set using an acceptor-only and donor-only sample. Analysis showed evident heterogeneity and G1 cell cycle dependence (**Figure 4D**), with a FRET efficiency of E=8±3 % (p<0.03); the interaction profile was similar for S and G2 phases, E=5±3 %. This was further confirmed by FLIM-FRET imaging, which is represented in **Figure 4B**; each cell was followed over multiple cell cycles using flexible ROIs to account for cell movement and distortion over time. Donor lifetimes were measured using the multi-exponential decay model, and chi-square fitting and Instrument Response Function (IRF) were used to determine the goodness of the data. Lifetime values were plotted in the analysis, showing a significant correlation between interaction and cell cycle phases. G1 cells showed a Bax lifetime of 2.2±0.3 ns, and NonG1 showed 2.35±0.3 ns, indicating a stronger interaction in the G1 phase (*p*<0.0001; **Figure 4E**). When we performed the FRET efficiency calculation with lifetime data from **Figure 4E**, the results showed strong interaction in G1 and comparatively weaker interaction in non-G1 phases, as seen in **Figure 4F**. Finally, we performed cell cycle-specific co-immunoprecipitation, which is represented in **Figure 4C **and Figure S2E; target Bcl-xL was pulled using Bax as bait and probed with anti-Bcl-xL antibody. As shown in the figure, non-G1 phases showed moderately lower interaction pulldown of YFP Bcl-xL. This highlighted that subtle interaction heterogeneity was indistinguishable in Co-immunoprecipitation due to the large number of cells in samples, normalizing the heterogeneity of interactions in the cells.

### Cellular stress and Bax activation phase are marked by strong interaction between ECFP Bax-YFP Bcl-xL

**Figure 5:**
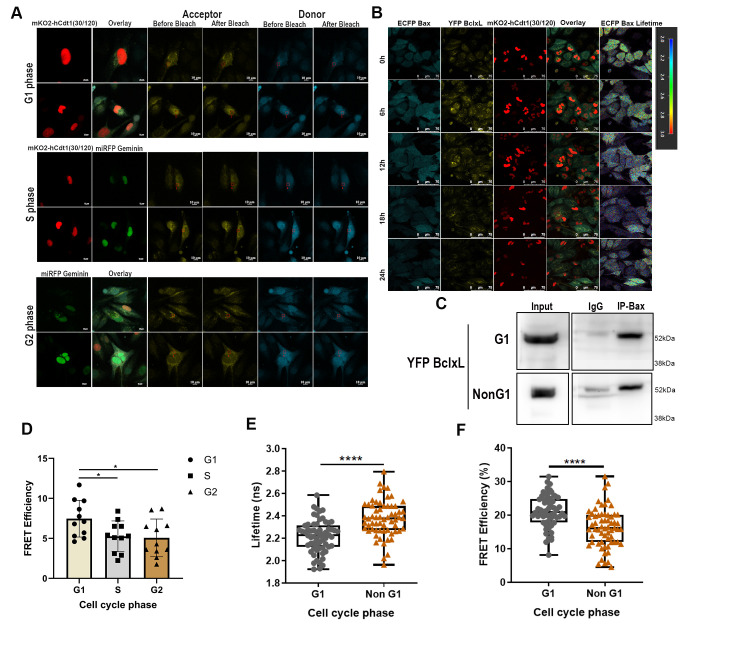
FIGURE 5: ECFP Bax and YFP Bcl-xL show increased interaction in cell stress and Bax activation. **(A)** FLIM-FRET imaging analysis of ECFP Bax lifetime to assess interaction was performed after treatment with chemotherapeutic drugs; images represent ECFP Bax lifetime heatmap (scale 2-3 ns). **(B) **represents a statistical analysis of FLIM-FRET imaging from **(A), **ECFP Bax lifetime after stress induction is analyzed in both G1 and non-G1 phases, showing robust correlation between stress and strong interaction (n=30) ****p<0.0001 (one-way/two-way ANOVA). Similar imaging was done to understand Bax accumulation and interaction during cell stress by analyzing the ECFP Bax lifetime of Bax accumulated on the mitochondrial membrane **(C)**, marking a crucial Bax activation event; arrows represent areas of Bax activation in wild-type and mutant cells (scale 2-3 ns)**. (D) **shows ECFP Bax lifetime analysis from FLIM-FRET imaging from **(C)**; Bax activation causes a strong interaction between ECFP Bax-YFP Bcl-xL when analyzed for a reduction in ECFP lifetime in wild-type cells. In contrast, mutants show no such changes (n=15) ****p<0.0001 (student’s t-test). **(E)** We quantified the ECFP Bax accumulation over time after treatment with resveratrol, as the Bax puncta formation increased, the corresponding lifetime decrease was visualized. **(F)** represents analysis of puncta formation and associated Bax lifetime decrease with time. **(G)** and **(H) **show FRET AB interaction analysis between ECFP Bax-YFP Bcl-xL under cell stress; cells were categorized into stress and Bax-activated stage based on Bax accumulation. The analysis in **(H)** shows FRET efficiency changes of cell stress conditions (n=15) ****p<0.0001 (one-way ANOVA). **(I) **The co-expressing ECFP Bax-YFP Bcl-xL cells were treated with two BH3 mimetics, A-1331852 and A-1155463, to analyze the effect of mimetics on interaction. **(J) **As shown, a significant increase in ECFP Bax lifetime was observed in cells (n=25) ****p<0.0001 (one-way ANOVA).

Beyond protein expression-dependent cell death and survival decisions by pro-death and anti-death factors, the decisive role of the interaction dynamics in shaping cell death is yet to emerge. However, there have been contradicting notions related to the interaction between Bax and Bcl-xL and its outcome in the progression of cell death. In the crucial step of cell death, activation/accumulation of Bax on the mitochondrial membrane and pore formation leads to cytochrome C release 24. We have used FRET and lifetime imaging to study the interaction dynamics after cells were exposed to apoptosis-inducing stress using Gossypol and Paclitaxel. As shown in **Figure 5A**, the cells showed distinct interaction status under the stress conditions. Upon ECFP Bax lifetime analysis, Gossypol-treated cells showed stronger interaction and consequently higher cell death, but Paclitaxel-treated cells showed reduced basal interaction and better overall survival (**Figure 5B**). The complete 48 h time-lapse video of stress-induced ECFP Bax lifetime analysis is shown in video S4**. **The interaction was affected in all cell cycle phases, with strong interaction increasing in both G1 and non-G1 phases upon stress induction based on donor lifetime data. The results point towards a mechanism where basal interaction between partners is vital for survival. Consistent with this notion, the opposite effect was observed in most weakly interacting cells in fate tracing. Bax activation is an event where cytosolic Bax accumulates on the mitochondrial membrane, triggering its permeabilization and apoptosis 25. We have analysed the Bax activation from puncta formation and corresponding lifetime changes in ECFP Bax cells, expressing wildtype Bcl-xL and interaction defective mutants after resveratrol treatment (**Figure 5C**). A significant reduction in lifetime was seen in wildtype cells, predominantly at mitochondria, indicating a strong interaction in cells. At the same time, the mutants did not show any noticeable lifetime change, which was a sign of abrogated interaction between proteins (**Figure 5D**). White arrows in the figure show the areas where activated Bax can be seen in cells with Mitotracker Deep Red co-localizing with proteins. From analysis, it was clear that the activated region showed a lifetime of 1.96±0.08 ns (E= 24.93±2.89%) in wildtype, while the mutant Y340K showed 2.58±0.1 ns and G377A showed 2.6±0.2 ns, in activated conditions. While it was true that Bax activation resulted in strong interaction between proteins in wildtype, a higher cell death was observed in the mutant FRET pair-containing cells, possibly resulting from Bax overexpression and nonfunctional mutants, making cells more prone to cell death. This aligns with previous findings that Bcl-xL could recruit Bax onto the mitochondrial membrane during stress 26,27. Further, we analyzed the formation of Bax activation puncta in cells over time and changes in the ECFP-Bax fluorescence lifetime. As the cells showed a higher accumulation of Bax, a higher reduction in ECFP Bax lifetime could be visualized, suggesting a strong interaction increase over time (**Figure 5E, F**)**.** As an additional proof of interaction increase during Bax activation, Acceptor photobleaching FRET was performed on cells after treatment with Gossypol and Resveratrol. We observed an increased interaction in treated cells by acceptor photobleaching FRET, as shown in **Figure 5G**. While the control FRET efficiency was E=7.22±0.58 %, treated cells showed a higher FRET efficiency, E=9.33±1.02 % (*p*<0.01). It was also seen that Bax-activated cells showed higher interaction between proteins compared to both control and treated cells, with FRET efficiency E 13.3±1.1% (p<0.0001) **Figure 5H**, thus cementing our observation that Bax-activated cells display higher interaction between proteins. Additionally, BH3 mimetics were used to assess the interaction changes and cell fate in U251 ECFP Bax-YFP Bcl-xL cells. Two selective Bcl-xL inhibitor mimetics, A-1155463 and A-1331852, were used at 100 nM each. High-resolution endpoint images were taken with Mito-Tracker Deep Red after 12 h of mimetic treatment (**Figure 5I**). The results showed a loss of interaction between proteins with lifetimes of 2.60±0.2 ns for A-1331852 and 2.55±0.1 ns for A-1155463 (**Figure 5J**).

## DISCUSSION

Tissue homeostasis requires coordinated signaling to regulate cell division and cell death. Because of the requirement for tight coordination, significant crosstalk exists between the cell cycle and cell death. The cell cycle is controlled by spatiotemporal changes in unique gene transcription, translation, protein degradation, and protein modification 28,29. In the last few years, intensive work has been done to understand cell cycle-dependent changes in gene transcription, protein abundance, localization, and protein modification 30,31. This has revealed that cell cycle progression events depend on gene transcripts and insight into the cell-to-cell variability of protein expression and response heterogeneity among genetically identical cells 32. A recent study of single-cell RNA sequencing using the FUCCI cell cycle probe enabled the identification of cell cycle-dependent transcriptional regulation that has helped to create cell cycle-dependent transcriptome and proteome 33. The study led to the identification of 530 genes that show variance and their correlation with the cell cycle. Interestingly, many proteins show cell-to-cell variation in a cell cycle-independent manner, too 33. This indicates the possible stochasticity factors or cellular states and functions governed by protein-protein interactions or interaction-dependent half-life of proteins. Studies have shown that, in addition to transcriptional regulation, many fast-progressing signaling events are regulated by changes in protein-protein interaction in space and time 17. Recent innovations in single-cell studies, such as single-cell transcriptomics, imaging, proteomics, single-cell sorting, etc, have provided valuable information about spatiotemporal alterations of proteins at the single-cell level to get insight into many pathological conditions 34,35. Similarly, there are highly sensitive approaches to studying the protein-protein interaction in live cells 36. In the current work, we have utilized FLIM-FRET along with the cell cycle indicator probe FUCCI to study the interaction between Bax and Bcl-xL at the single-cell level, along with cell cycle progression.

Even though the Bax-Bcl-xL interaction using FRET has been confirmed in a previous study 37, the implication of the interaction in the cell cycle and cell death is yet to be revealed. Previous studies used transient over-expression that often induces artifacts of localization, protein-protein interactions, and altered cell cycle. Our primary task was to create a stable expression system with both Bax and Bcl-xL using FRET donor and acceptor partners. The expression of Bax with donor fluorescent protein ECFP retained its pro-apoptotic activity and sensitized the cells to multiple drugs. Similarly, Bcl-xL demonstrated its anti-apoptotic functions. We have further confirmed that both proteins demonstrated their cell cycle regulatory functions in unperturbed cells alone and in combination. Further, to enable cell cycle visualization, we have utilized mKO2-hCdt1(30/120) from the original FUCCI as a G1 probe and the near-IR fluorescent version of Geminin. Co-expressing all four probes enables visualization of protein interaction dynamics and cell cycle in real-time, revealing the potential utility of the cell system to study macromolecular interactions during cell cycle and cell death.

A recent study from our lab demonstrated cell cycle-dependent redox alterations at mitochondria and cytosol 38. Similarly, we have demonstrated an interaction between p21 and Nrf2 as the key factor in tumor stem cell-dependent resistance 39. These experimental results and an increasing number of cell cycle-dependent proteomes suggest that defined protein expression and spatiotemporal changes in protein-protein associations further provide an additional layer of regulation for the tight control of cell cycle and cell death decisions.

In the current study using FLIM-FRET, we show that both Bax and Bcl-xL proteins maintain a basal interaction in general, but the interaction is much stronger in the G1 phase of the cell cycle. In cells with Bax and Bcl-xL overexpression, Bax-dependent acceleration in cell cycle progression took the upper hand over the cell cycle inhibitory function of Bcl-xL, as shown in **Figure 3D**. We have demonstrated that Bcl-xL is a dominant partner controlling cell cycle progression in G1 and S (**Figure 3C**), which could explain the stronger association of these proteins at G1. Previous studies have already linked the enhanced survival of cells at the G1 phase to the other phases 40.

However, under cell stress conditions, the role of the cell cycle takes a backseat as a strong interaction is seen irrespective of the cell cycle phase (**Figure 5A-D**). The results further demonstrate that a crucial event like Bax activation provides a base for stronger interaction between the proteins **Figure 5C-H**). Multiple reports place Bax activation/accumulation as a point of no return with Bax homo and hetero oligomerization with tBid as a catalyst for aggravating cell death 41,42. It is unclear whether anti-apoptotic members of the BCL2 family play some role in this; few reports indicate that Bax recruitment and activation can be mediated by BCL2 43.

Earlier studies by the David Andrews group have demonstrated that the Bcl-xL complex can bind to both c-bid and bad concurrently; however, c-bid-bound Bcl-xL is an activation switch for Bax 27. Recent work by the group further demonstrated the potential application of quantitative FAST FLIM to screen BH3 mimetic drugs by measuring drug effects on binding affinities of interacting Bcl2 family protein pairs 44. Earlier studies using FLIM-FRET further confirmed that the two binding interfaces enable Bim to double-bolt lock Bcl-xL and Bcl-2 in complexes resistant to displacement by BH3-mimetic drugs 45. Similarly, it has been shown that Bcl-xL binds to Bax after membrane embedding 46. These studies are consistent with our observation of increased interaction of Bcl-xL and Bax after translocation and activation on the mitochondrial membrane. Further studies are required to reveal the influence and dynamics of this retrotranslocation or acceleration towards ultimate cell death, enabling complete cytochrome c release.

Further studies using this approach and mitochondrial permeabilization events could address the influence of stronger interaction at mitochondria in shaping the progression of cell death. Similarly, additional studies using other interaction partners of cell death and cell cycle are possible using the approach for systematic analysis of diverse sets of regulators and the dynamics of their interaction in driving cell cycle-dependent response heterogeneity. One limitation of the approach is the need for highly monoclonal, stable cells to have noise-free data; the development process is time-consuming. However, once such systems are developed, defined studies using this approach would help create cell cycle-dependent and independent interactomes that could be useful for addressing challenging cell biology questions such as cell-to-cell variability in response heterogeneity and fractional cell killing by anticancer drugs.

## MATERIAL AND METHODS

### Cell cultures

The Glioblastoma multiforme cell line U251 MG was procured from the central cell repository of Rajiv Gandhi Centre for Biotechnology and used within ten passages after revival. The cells were maintained in a humidified CO_2_ chamber (5%) at 37°C with 10% fetal bovine serum (Thermo Fisher Scientific, A5670701, Origin- United States) and 1% antibiotics (Thermo Fisher Scientific, 15240062) added to DMEM (Thermo Fisher Scientific, 11965092) media.

### Expression vectors, cell transfection, and stable cells development 

Four expression vectors were used to develop a cell system for the simultaneous visualization of protein-protein interactions and cell cycle phases. To develop cells expressing interacting FRET partners, the U251 cell was co-transfected with 5 µg of ECFP Bax and YFP Bcl-xL plasmids using Lipofectamine^™^ 2000 (Thermo Fisher Scientific, 11668030). Both ECFP Bax (23) and YFP Bcl-xL (24) were kind gifts from Prof. Richard J Youle. The transfected cells were maintained under 800 µg/mL of G418 sulfate (Thermo Fisher Scientific, 10131015) selection until multiple colonies with distinct transgene expression were visible. The cells were further sorted to get individual cell populations with homogeneous levels of ECFP Bax, YFP Bcl-xL, or both using FACSAria III (Becton Dickinson, USA). The sorted cells were further subjected to colony selection to get single-cell colonies stably expressing ECFP Bax only, YFP Bcl-xL only, and both ECFP Bax-YFP Bcl-xL. Later, cells with ECFP Bax, YFP Bcl-xL, and both ECFP Bax-YFP Bcl-xL were then used to generate FUCCI based cell cycle indicator probe. Briefly, the respective cells were first electroporated with 5 µg of G1/G1/S probe mKO2-hCdt1(30/120) using a Neon transfection system (Thermo Fisher Scientific, MPK1096). The monomeric Kusabira orange with hCdt1(30/120) as G1/G1S reporter, the mKO2-hCdt1(30/120) / pCSII-EF was a kind gift from Dr. Atsushi Miyawaki from the RIKEN BRC (21). We have utilized miRFP-based S/G2/M indicator probe miRFP670v1-hGem(1/110) (miRFP Geminin hereafter) to generate complete Cell cycle indicator FUCCI in ECFP Bax-YFP Bcl-xL- mKO2-hCdt1(30/120) stable cell colonies by second electroporation. The plasmid pCSII-EF-miRFP670v1-hGem(1/110) (22) was a gift from Vladislav Verkhusha (Addgene plasmid # 80006; http://n2t.net/addgene:80006; RID:Addgene_80006). miRFP hGem(1/110) protein was used to aid imaging of hCdt1(30/120) along with ECFP-YFP using conventional confocal multicolor imaging using 440 (405) /488/562/640 laser combinations.

### Bcl-xL mutants generation and transfection

We generated a wildtype and two mutants by inducing point mutations in Bcl-xL. The Y101K mutation in BH1 has been described for its inability to bind to BH3 proteins earlier 47. We generated the mutant with a Venus N-terminal tag, resulting in a Y340K (Venus-Bcl-xL Y340K) mutation in the hybrid construct. Furthermore, another Bcl-xL mutant was generated with a G138A mutation, known to render the protein functionally inactive 48. Similar to Y340K, this mutant was also tagged with the Venus N-terminal tag, resulting in a G377A (Venus-Bcl-xL G377A) mutation in the hybrid construct. Wildtype (Venus-Bcl-xL) was generated similarly with a Venus N-terminal tag in Bcl-xL. These constructs were transfected into ECFP Bax-expressing cells separately using Lipofectamine transfection as described above. The cells were then maintained in Puromycin selection (Thermo Fisher Scientific, A1113803) for a few days, and colonies were picked for further experiments.

### Functional validation by imaging and Annexin-BFP FACS

The single-cell expanded colonies with fluorescent proteins were used for imaging experiments. For the functional validation of the stable colonies, real-time imaging was carried out for 36 hours after exposing them to stress-inducing compounds. Chromatin condensation through 5 µM Hoechst-33342 (Thermo Fisher Scientific, 62249) binding was used for quantitative readout of cell death. Quantitative analysis of apoptosis was carried out using recombinant Annexin V BFP as the marker of apoptosis (synthesized in the lab) by flow cytometry. The list of compounds used in experiments are mentioned in **Table 1**.

**Table 1:** Compounds used in the study.

**Sl.No.**	**Compounds**	**Working concentration**	**Mechanism of action**
1	Gossypol (Sigma-Aldrich, G8761)	10 µM	Induces cellular ROS, resulting in cell death 49
2	Resveratrol (Sigma-Aldrich, R5010)	25 µM	Induces Bax activation through action on XIAP, Bid, and t-Bid as well 50
3	Paclitaxel (Sigma-Aldrich, T7191)	1 µM	Induces microtubule stabilization, causing cell cycle arrest 51
4	Raptinal (Sigma-Aldrich, SML1745)	5 µM	Rapid induction of MOMP, initiating intrinsic apoptosis 52
5	A-1155463(MedChemExpress, HY-19725)	100 nM	A strong selective Bcl-xL inhibitor 53
6	A-1331852(MedChemExpress, HY-19741)	100 nM	Causes selective disruption of the Bcl-xL complex with BH3 proteins 44

### Cell cycle synchronization 

Cell cycle synchronization was performed with Thymidine (Sigma-Aldrich, T9250) using a double thymidine block as described by Chen *et al.* 54. Briefly, thymidine-containing media was added to the cells at a 2 mM concentration, and the first block was given for 18 h; a brief release was done for 9 h, followed by a second block for another 18 h. This resulted in synchronized cells stuck at the G1/S boundary. The cell cycle analysis was performed at three time points from 0 h, 4 h, and 8 h, respectively, to assess the cell cycle by FACS. For this, the single cells prepared were stained with 5 µM Hoechst 33342 (Thermo Fisher Scientific, H3570) for 10-12 minutes with periodic tapping in the dark and analyzed with a standard DAPI filter set at 355 nm excitation (FACS Aria Fusion, SORP, Becton Dickinson, USA). Doublet discrimination was done against the DAPI width signal vs the DAPI area.

### Acceptor photobleaching FRET (FRET AB)

Acceptor photobleaching FRET was used as an intensity-based tool to detect the interaction between Bax and Bcl-xL. The cells were seeded in an 8-well glass-bottom chamber at 50-60% confluency. After overnight attachment and growth of cells, the glass bottom chamber was mounted on a Zeiss LSM 980 Airyscan 2 Confocal Laser Scanning Microscope (Carl Zeiss NTS Ltd., Germany) with an incubator CO_2_ chamber (5%) at 37°C compatible with microscopy. The donor-only and acceptor-only containing cells were used to set imaging conditions and assess the post-bleaching behaviour of fluorophores. ECFP was excited with a diode 445 nm laser (8-10% laser power) to collect an emission range of 460-495 nm; similarly, YFP was excited with a 514 nm laser (8-10% laser power), and emission was collected at 525-550 nm.

A dry 40x objective with NA 0.95 was used for quick acquisition of the bleached area. The acceptor (YFP Bcl-xL) was bleached with a diode laser-514 nm at 80% laser power for 20 iterations, resulting in a loss of FRET between the proteins. Distinct regions of interest (ROI) were defined within multiple cells for photobleaching to calculate the effective increase in the intensity of donor (ECFP Bax) from pre-bleach readings. The FRET efficiency was measured using the formula mentioned below.

E= (1- (*I*_0_/*I*_t_)) × 100

Where *I*_0_ represents ECFP Bax intensity before bleach and *I*_t_ represents ECFP Bax intensity after bleach.

For imaging the cell cycle indicator, mKO2-hCdt1(30/120), a 555 nm laser (12-15% laser power) from a white light source was used, collecting an emission range of 560-600 nm, and miRFP Geminin was excited with a 639 nm laser (15% laser power), collecting emission of 660-720 nm using specific spectral detectors of SP8 WLL confocal microscope (Leica Microsystem, Germany).

### Western blotting, GFP Trap, and Co-immunoprecipitation

The cells were seeded in 60 mm and 100 mm culture dishes. After attaining 80% confluency, cells were scraped with ice-cold PBS (pH 7.4) and lysed in IP lysis buffer (50 mM HEPES, pH 7.4, 100 mM NaCl, 1 mM EDTA, 1% Nonidet P-40, protease inhibitor cocktail and phosphatase inhibitor cocktail) for immunoblotting. The transfected proteins were probed using specific antibodies such as anti-GFP (Cell Signaling Technology, 2956), anti-Bax (Santacruz, 493), anti-Bcl-xL (Cell Signaling Technology, 9941), and anti-GAPDH (Cell Signaling Technology, 2118). Anti-GFP, anti-Bcl-xL, and anti-GAPDH antibodies were used at a 1:1000 dilution, while anti-Bax was used at a 1:500 dilution. The HRP-conjugated secondary antibodies anti-Rabbit (Cell Signaling Technology, 9302) and anti-Mouse (Sigma-Aldrich, A0168) were used at 1:10000 dilutions. GFP Trap co-immunoprecipitation assay was performed to validate the interaction between Bax and Bcl-xL using the whole-cell lysates of U251 YFP Bcl-xL cells to establish the interaction between YFP Bcl-xL and endogenous Bax. The co-immunoprecipitation was done using a ChromoTek GFP-Trap® Magnetic Particles M-270 Kit (Proteintech Inc., USA) as per the manufacturer's instructions. For cell cycle-specific CO-IP, U251 ECFP Bax YFP Bcl-xL cells were synchronized using a double thymidine block as described. Cells were released and harvested at specific time points for maximum cell cycle phase-specific populations. Key to this was using the synchronization data to identify exact time points for harvesting maximum cells in particular phases. Cells were further lysed for 1 h at 4°C in lysis buffer (50 mM HEPES, pH 7.4, 100 mM NaCl, 1 mM EDTA, 1% Nonidet P-40), protease inhibitor cocktail, and phosphatase inhibitor cocktail. The lysates were incubated with Anti-Bax antibody overnight at 4°C with end-over-end mixing. The complex was pulled down using Pierce^™^ Protein G Agarose (Thermo Fisher Scientific, 20399) pre-equilibrated in lysis buffer for 2 h. The beads were washed thrice with lysis buffer, followed by boiling in 1x Laemmli buffer. The samples were analyzed by immunoblotting with HRP-conjugated primary antibodies. Co-immunoprecipitation was also performed in ECFP Bax-YFP Bcl-xL (WT) and mutants expressing cells through Bax IP, with the protocol followed as described above.

### Laser scanning confocal Fluorescence lifetime imaging

The Leica SP8 FALCON (FAst Lifetime CONtrast) microscope (Leica Microsystems, Germany) was used to perform real-time fluorescence lifetime imaging to study the interaction variations between ECFP Bax-YFP Bcl-xL. For real-time lifetime imaging, the cells were counted and seeded in 8-well glass-bottom chambers, mounted, and imaged on a microscope for 24-48 h. The cells were maintained on an onstage incubator (BRICK) system (Life Imaging Systems, Switzerland) to maintain 5% CO_2_ and 37°C temperature. Time-correlated single photon counting (TCSPC) measures photon arrival time to the detector after pulsed excitation, and the incremental counts are used to build up a lifetime histogram. Pulsed laser excitation works best for detecting the exponential decay of fluorophores after short excitation pulses from the laser. The signals were acquired sequentially; ECFP Bax excitation was achieved using a 445 nm pulsed laser diode (40 MHz repetition rate; pulse width ~100 ps). A TCSPC setup equipped with single-photon sensitive hybrid PMA detectors was utilized for the detection of emission. Emission was detected through the 475-500nm range detection. The dry 40x objective with NA 0.95 was utilized for time-lapse multipoint imaging and endpoint imaging. The laser power for the pulsed laser was 8%, the frame repetition for acquisition was 10-15, and the single-cell images were acquired at 512 x 512 resolution. The median detection of 300-500 photons/pixel was achieved for various experiments. It was kept in this range to avoid photobleaching-induced artifacts in long-term time-lapse imaging.

The lifetime measurement was performed using the pre-set mode for FLIM in LasX software. Donor alone and both donor-acceptor samples were used for measuring lifetime in FLIM mode, and then the lifetime and FRET analysis were done in FRET mode of LasX, using the unquenched donor lifetime value as a control. IRF and exponential decay were determined using the best fit with a double exponential decay model for the donor alone, and further analysis with the best fitting exponential decay model for both donor-acceptor lifetime images. The relative distances, FRET, and binding affinity were calculated after the analysis. Mean ROI analysis was performed by drawing ROIs on cell boundaries and tracking them throughout the real-time imaging experiment. Lifetime was recorded separately for each ROI, and fitting was done separately for each. The resulting FRET efficiency was calculated using the standard formula:

E= (1- (β_m_^DA^ /β_m_^D^)) × 100

Where β_m_^D^ represents ECFP Bax mean lifetime(ns) in U251 ECFP Bax only cells and β_m_^DA^ represents ECFP Bax mean lifetime(ns) in U251 ECFP Bax YFP Bcl-xL cells.

### Statistical analysis

All statistical analyses were performed using GraphPad Prism 8.0.2. Student’s t-test, One-way ANOVA, and Two-way ANOVA with multiple comparisons were performed depending on the variables in the dataset. *P*<0.05(*) was considered significant, with increasing significance with each decimal. Analyses were performed assuming a normal distribution of data in the dataset.

## CONFLICT OF INTEREST

The authors declare no conflict of interest.

## SUPPLEMENTAL MATERIAL 

Click here for supplemental data file.

Click here for supplemental data file.

Click here for supplemental data file.

Click here for supplemental data file.

Click here for supplemental data file.

All supplemental data for this article are available online at https://www.cell-stress.com/researcharticles/2025a-halikar-cell-stress/
