# Supplementary material for: Bax- Bcl-xL interaction dynamics during the progression of cell cycle and cell death using FLIM-FRET
Source: Cell Stress. 2025 Jul 8;9:143–57. doi: 10.15698/cst2025.07.307 (PMC12285600; doi:10.15698/cst2025.07.307)
Supplement: Supplementary file 1 [file ces-09-143-s01.pdf]

Supplemental Material

Bax- Bcl-xL interaction dynamics during the progression of cell cycle and cell death using FLIM-FRET

Aman Munirpasha Halikar[1,2], Aneesh Chandrasekharan[1], Asha Lekshmi[1], Aswathy Sivasailam[1], Jain Tiffee P J[1,2], Shivanshu Kumar Tiwari[1,2], Aijaz Ahmad Rather[1,2] and TR Santhoshkumar[1,*]

[1] Cancer Research Program, Rajiv Gandhi Centre for Biotechnology, Poojappura, Thycaud P.O., Thiruvananthapuram, Kerala 695014, India.
[2] Manipal Academy of Higher Education (MAHE), Manipal, Karnataka-576104, India.

* Corresponding Author:

T. R. Santhoshkumar, Ph.D, Cancer Research Program-1, Rajiv Gandhi Centre for Biotechnology, Poojappura, Thycaud P.O., Thiruvananthapuram, Kerala 695 014, India; Phone: +91- 471-2529400; Fax: + 91- 471-2348096; E-mail: trsanthosh@rgcb.res.in

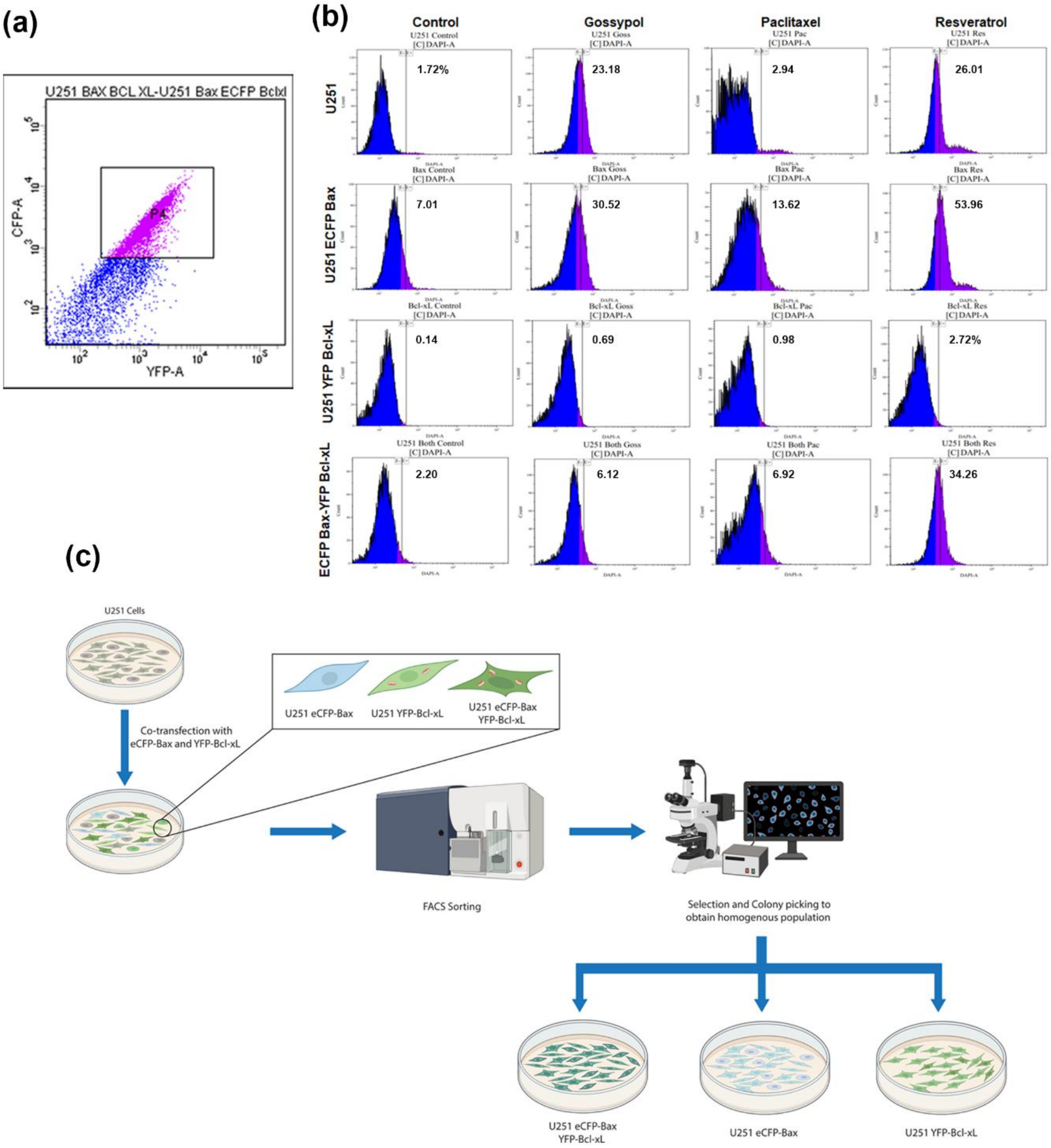

Supplemental FIGURE S1 ● FACS sorting gating parameters for developing U251 ECFP Bax-YPF Bcl-xL cells is shown in (a). Annexin V BFP analysis for cell death induction in differetn protein expressin cells, detailed data is shown in (b). The schematic illustration of stable ECFP Bax, YPF Bcl-xL, and ECFP Bax- YPF Bcl-xL FRET parnter cell development is shown in (c).

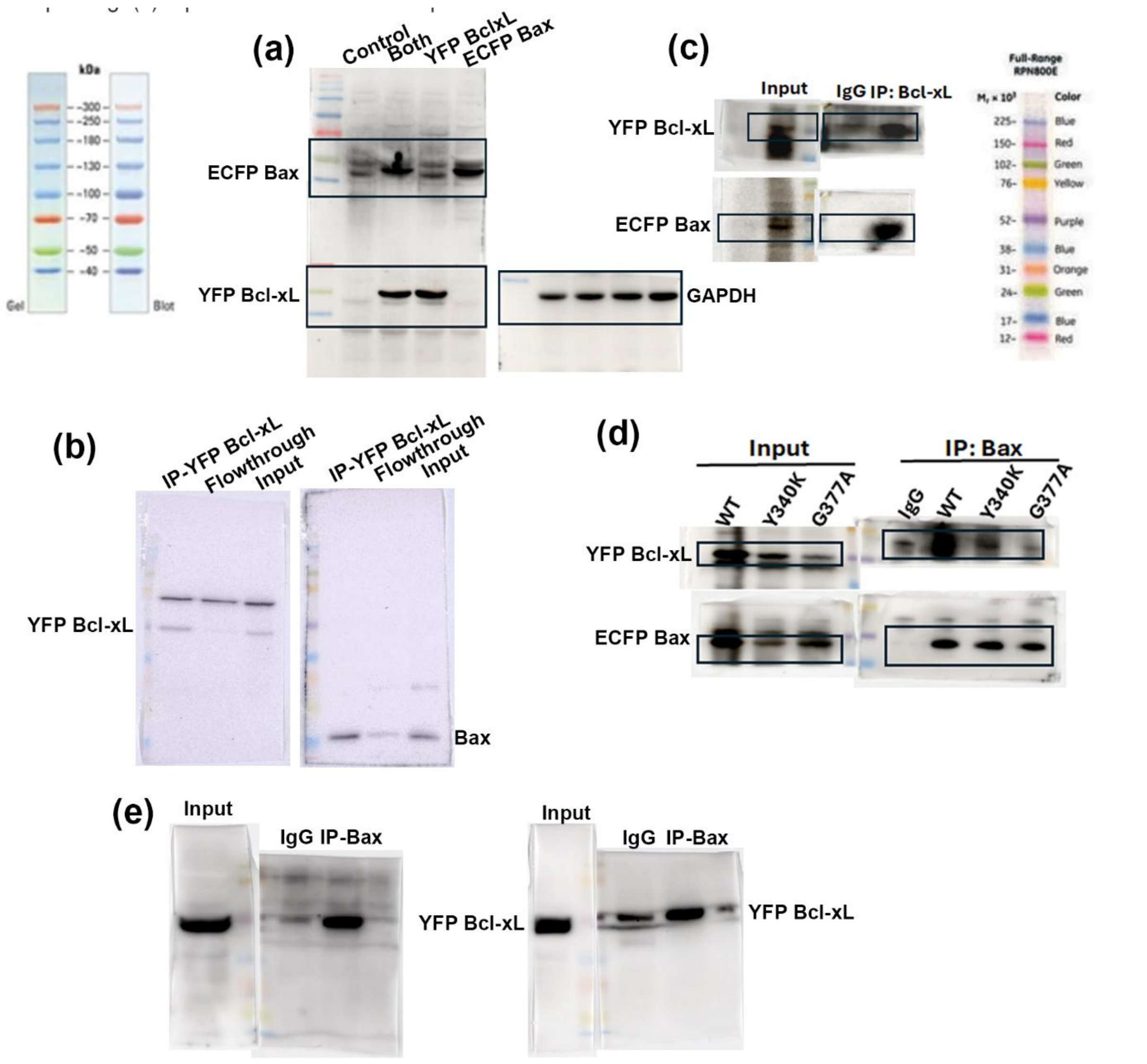

Supplemental FIGURE S2 ● Uncropped immunoblots of alle experiments are shown with Rainbow marker for reference, rectangular shapes show the area of interest that was cropped for main figures. Uncropped blots of protein expression validation from Figure 1D are shown in **(a)**; ECFP Bax and YFP Bcl-xL refer to ECFP Bax and YFP Bcl-xL expressing cells, while both represent co-expressing cells, respectively. Similarly, uncropped IP blots of interaction between proteins from Figure 2 are shown; **(b)** shows GFP Trap CO-IP immunoblots of Figure 2E, and **(c)** shows uncropped physically cut CO—IP blots of Figure 2F. Uncropped physically cut CO-IP immunoblots from Figure 2G of IP wildtype against mutants is represented in **(d)**, blots had to be cut due to proximity of both protein sizes making it difficult for IP probing. **(e)** represents IP blots for comparison IP between G1 and NonG1 cells.

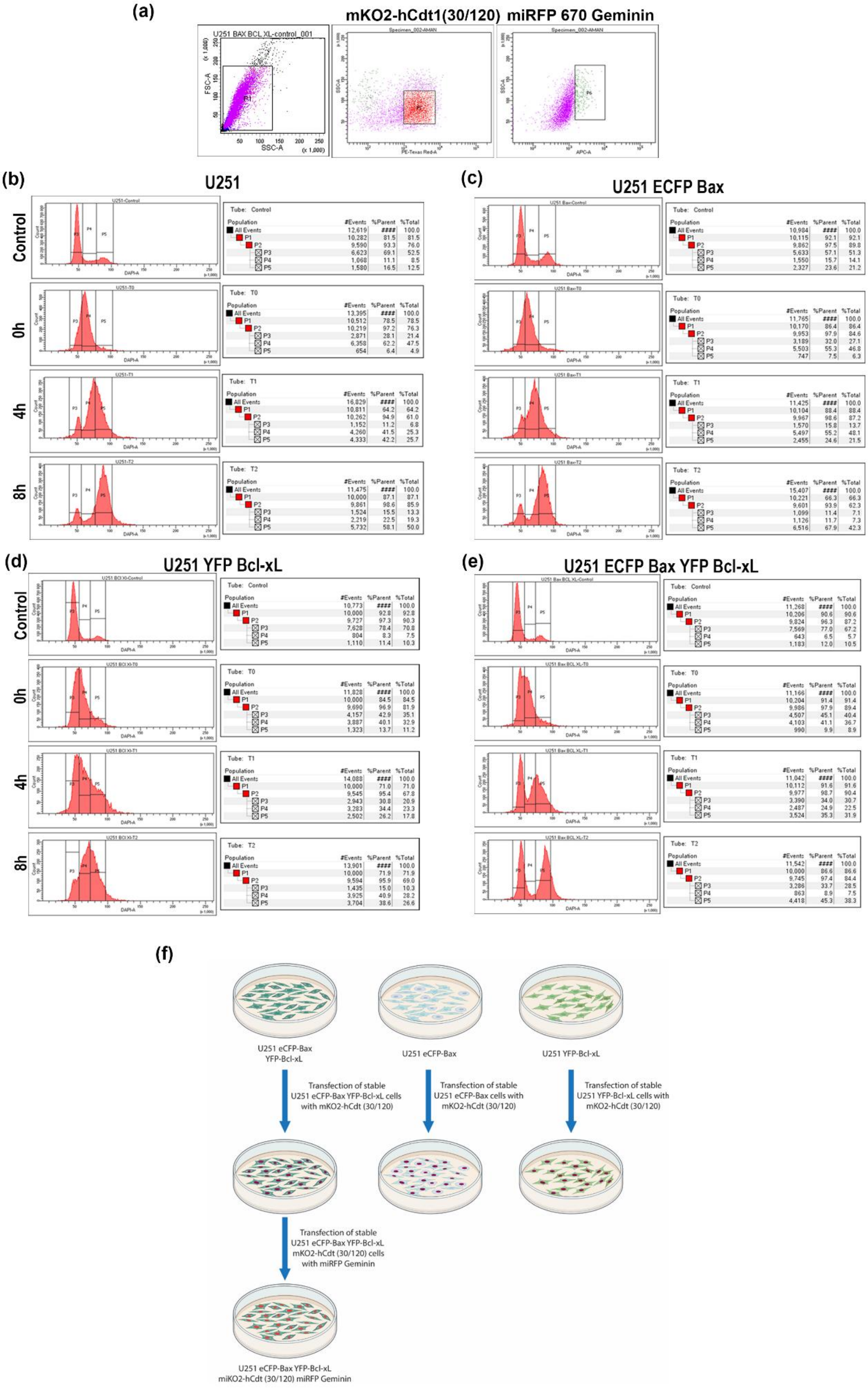

Supplemental FIGURE S3 ● FACS sorting gate used for selection of mKO2-hCdt(30/120) and miRFP Geminin cells for stable expression shown in (a). Detailed raw data of cell cycle analysis (Figure 3A-D) of stable cells is shown in (b) U251, (c) ECFP Bax, (d) YFP Bcl-xL, and (e) ECFP Bax YFP Bcl-xL. A schematic of the development of stable mKO2-hCdt(30/120)/FUCCI cells is shown in (f).

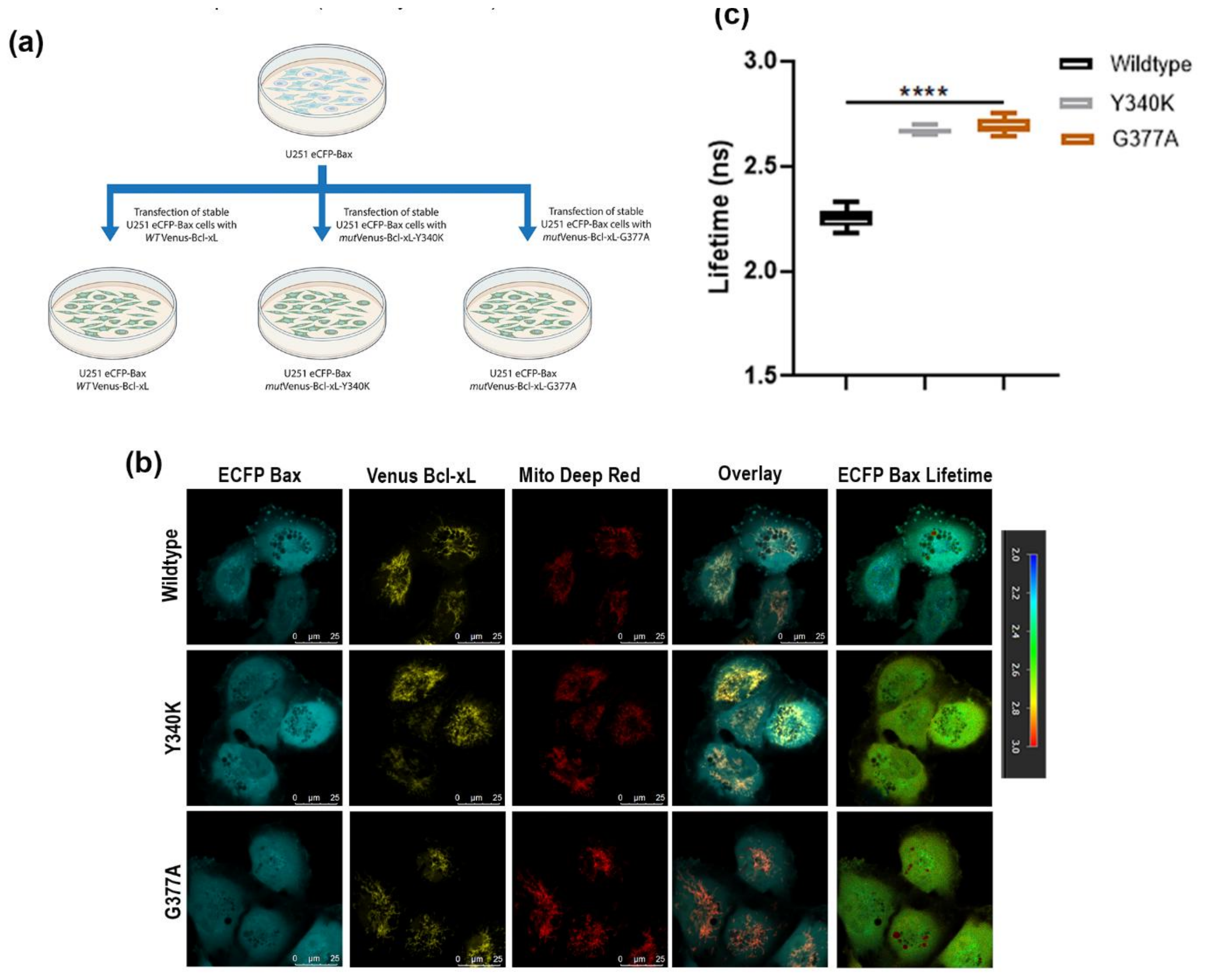

Supplemental FIGURE S4 ● Cells stably expressing ECFP Bax were transfected with 3 recombinant Bcl-xL constructs. Mutants were generated at Y101K and G138A, the mutants and a wildtype were also given an N-terminal fusion tag with Venus resulting in Venus Bcl-xL, mutVenus Bcl-xL Y340K, and mutVenus Bcl-xL G377A, respectively. (a) represents the schematic of cell development with Venus Bcl-xL constructs. (b) represents imaging data of FLIM imaging performed on Venus Bcl-xL wildtype and mutant cells. (c) The analysis represents FLIM analysis and comparison between Venus Bxl-xL wildtype and mutant cells. **** $p<0.0001$ (one-way ANOVA).

Supplementary videos

Video S1- supplementary video shows the progression of cell cycle and division in U251 ECFP Bax- mKO2-hCdt1(30/120) performed for 48h.

Video S2- supplementary video shows the progression of cell cycle and division in U251 YFP Bcl-xL- mKO2-hCdt1(30/120) performed for 48h.

Video S3- Time-lapse video of stable expression of cell cycle progression probes mKO2-hCdt1(30/120) **(Red)** and miRFP Geminin **(Green)** with ECFP Bax and YFP Bcl-xL **(Merged)** in the U251 MG cells **(Figure 3I)**.

Video S4- Time-lapse video of drug-induced cell stress lifetime imaging with FRET FLIM shown in **Figure 5A.** The time-lapse of control, Gossypol, and Paclitaxel-treated cells is shown in the video.
